# Tunnel Technique and Subepithelial Connective Tissue Graft, With or Without Cross-Linked Hyaluronic Acid, in the Treatment of Multiple Gingival Recessions: Prognostic Parameters for Clinical Treatment Outcomes of Randomized Controlled Trial

**DOI:** 10.3390/jcm13226758

**Published:** 2024-11-10

**Authors:** Bartłomiej Górski, Izabela Maria Skierska, Kacper Nijakowski, Aniela Brodzikowska

**Affiliations:** 1 Department of Periodontal and Oral Mucosa Diseases, Medical University of Warsaw, 02-097 Warsaw, Poland; iza.maria.skierska@gmail.com; 2 Department of Conservative Dentistry and Endodontics, Poznan University of Medical Sciences, 60-812 Poznan, Poland; kacpernijakowski@ump.edu.pl; 3 Department of Conservative Dentistry, Medical University of Warsaw, 02-097 Warsaw, Poland; aniela.brodzikowska@wum.edu.pl

**Keywords:** esthetics, hyaluronic acid, logistic regression, modified coronally advanced tunnel technique, multiple gingival recessions, subepithelial connective tissue graft

## Abstract

**Objectives:** This study aimed to investigate factors that influence the 12-month outcomes after the treatment of multiple gingival recessions (GRs) with a modified coronally advanced tunnel (MCAT) and a subepithelial connective tissue graft (SCTG), with cross-linked hyaluronic acid (HA, tests) or without (controls). **Materials and Methods**: Twenty-four patients with 266 GRs were treated. A logistic regression model was set to identify the baseline parameters that could predict the 12-month outcomes. The study protocol was registered at ClinicalTrials.gov (ID No. NCT05045586). **Results**: The evaluated clinical and esthetic parameters showed marked improvement in both groups without any statistically significant differences between the groups, with the exception of the soft tissue texture (STT). The STT was in favor of the HA group (0.96 versus 0.73, *p* = 0.0091). The likelihood of an MRC > 85%, of achieving CRC, and of gaining an RES = 10 was the highest for the incisors (reference group) and the lowest for the molars (OR = 0.046, *p* = 0.005). With each 1 mm increase in the baseline clinical attachment level, the odds of failure (MRC < 85%, not achieving CRC) increased, whereas each 1 mm increase in the baseline keratinized tissue width (KTW) improved the chances of an MRC > 85%, of achieving CRC, and of gaining an RES = 10. The application of HA increased the likelihood of a perfect RES more than twofold (OR = 2.683, *p* = 0.001). **Conclusions**: The application of HA improved the 12-month esthetic outcomes after the treatment of GRs with the MCAT technique. The baseline CAL, KTW, and tooth type predicted the 12-month MRC, CRC, and RES. An evaluation of the baseline characteristics of the surgical area might help clinicians develop individualized treatment plans.

## 1. Introduction

The apical migration of the gingival margin (GM) relative to the cementoenamel junction (CEJ) is described as a gingival recession (GR) [1]. GRs affect both younger and older populations, with a prevalence of up to 70% in individuals aged >50 years [2]. Based on the interproximal clinical attachment loss, GRs are classified into recession type 1 (RT1), with no loss of interdental attachment; recession type 2 (RT2), when the interdental attachment loss is smaller than the buccal attachment loss; and recession type 3 (RT3), when the interdental attachment loss is greater than the buccal attachment loss [3]. GRs may lead to an impaired esthetic appearance, suboptimal plaque control, caries and/or non-carious cervical lesions, and teeth hypersensitivity [4].

Several surgical techniques have been introduced for the treatment of GRs, with an autogenous subepithelial connective tissue graft (SCTG) being used as the “gold standard” [5]. A recent systematic review showed a high efficiency for the modified coronally advanced tunnel technique (MCAT), as evidenced by a mean root coverage (MRC) of 87.87 ± 16.45% for multiple GRs and of 82.75 ± 19.7% for single GRs [6]. Another network meta-analysis found that the tunnel technique was significantly associated with a higher root coverage esthetic score (RES) than a coronally advanced flap (CAF) (0.84 [95% CI = 0.15–1.53]; *p* = 0.01) [7]. Due to a limited flap extension and a lack of vertical incisions, an MCAT offers several benefits, such as improved blood supply and graft nutrition, faster healing, and reduced postoperative morbidity [7,8]. Even though multiple GRs anatomically and technically are more difficult to treat, an SCTG combined with an MCAT has been suggested as a safe, predictable, and highly effective surgical approach [9,10,11].

With the aim of improving soft tissue healing in GR treatments, different biomaterials have been studied, among which an acellular dermal matrix (ADM) graft, a porcine-derived collagen matrix (XDM), an enamel matrix derivative, a platelet-rich fibrin membrane (PRF), and hyaluronic acid (HA) attracted particular attention [12,13,14,15]. HA is a multifunctional natural biopolymer, a linear glycosaminoglycan (GAG) composed of disaccharides containing glucuronic acid and N-acetylglucosamine, which is a paramount constituent of the extracellular matrix [16,17]. It is well known for its antibacterial, antifungal, and anti-inflammatory properties, in addition to its angiogenic and homeostatic effects that enhance wound healing in a wide range of human tissues, including periodontal tissues [18]. A recently published systematic review on the additional benefit of HA in the surgical treatment of GRs concluded that there was an advantageous effect on the clinical outcomes in the short term [19]. The overall magnitude, however, was limited, owing to large heterogeneity among the surgical modalities, commercial formulations, and application methods of HA.

The favorable outcomes of periodontal plastic procedures bank on a plethora of various parameters. The possible factors potentially associated with the clinical outcomes after the surgical treatment of GRs can be divided into patient-related (plaque control, smoking, general health, compliance), local (recession height and width, presence of keratinized tissue, gingival thickness, loss of interproximal attachment, tooth type and tooth location, presence of scars and frenula), and surgical-related (flap design, root surface biomodification, type of graft, flap tension) [20,21,22]. All of these factors should be taken into account in the clinical setting. To the best of the authors’ knowledge, no studies have yet evaluated site-specific characteristics that might predict the results after the root coverage of multiple RT1 and RT2 recessions with an MCAT, an SCTG, and HA. Therefore, the aim of this article was to identify potential preoperative predictors of the clinical and esthetic outcomes 12 months after the treatment of multiple GRs with an MCAT, an SCTG, and HA.

## 2. Materials and Methods

### 2.1. Study Design

This study was conducted as a split-mouth, double-blinded, controlled trial (RCT). It was carried out in accordance with the Declaration of Helsinki, and approved by the Ethics Committee of the Medical University of Warsaw (approval No. KB/119/2021). The study was registered in ClinicalTrials.gov (NCT05045586). All the patients signed informed consent forms prior to the treatment. The patients and researchers who participated in the study were blinded to the allocation of defects to the tested interventions.

This is a secondary analysis of the data derived from a study published elsewhere [23].

This manuscript was prepared in line with the CONSORT guidelines.

### 2.2. Sample Size Calculation

The sample size calculation applied prior to the recruitment was based on the primary outcome of the mean root coverage (MRC). The expected mean difference was 8.92% and the expected standard deviation was 7.39% per group [24]. Twelve subjects per group were required to detect an 8.92% difference between the groups, assuming 80% power and α = 0.05. Considering the availability of cases, the final sample size was decided to be 24 patients.

### 2.3. Study Endpoints

The primary endpoint was the mean root coverage (MRC) at 12 months [25]. The secondary endpoints were the GR reduction, the clinical attachment level (CAL) gain, the keratinized tissue thickness (KTW) increase, the gingival thickness (GT) increase, and the RES score [25].

### 2.4. Study Sample

**Figure 1:**
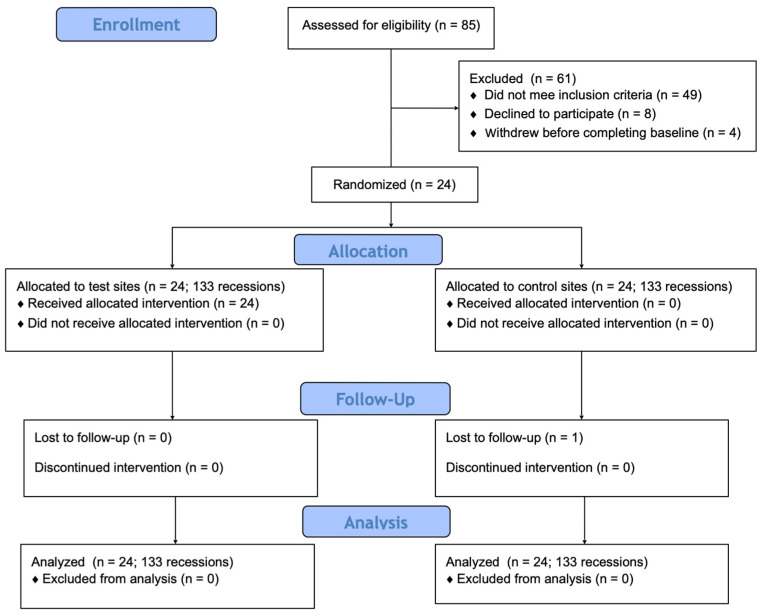
Consort diagram showing study design.

Subject recruitment started in April 2021 and finalized in May 2022 among patients referred to the Department of Periodontology and Oral Mucosa Diseases of the Medical University of Warsaw (Figure 1). Systematically healthy subjects who were eighteen years old or older were selected to participate in the RCT if they were diagnosed with multiple gingival recessions of RT1 and/or RT2 that were at least 1 mm deep with a detectable CEJ [3]. They had to demonstrate good oral hygiene (full-mouth plaque < 15% and full-mouth bleeding on probing < 15%) [26]. The exclusion criteria were as follows: (1) gingival recessions of type III (RT3); (2) a systematic disease that compromises wound healing or hemostasis (e.g., tumors, cardiovascular diseases, uncontrolled diabetes mellitus); (4) infectious diseases (hepatitis, tuberculosis, HIV); (4) caries lesions or restorations in the cervical area; (5) untreated periodontal conditions; (6) the use of medications affecting periodontal status (anti-inflammatory, antibiotic, anti-resorptive, or immunosuppressive medications, or phenytoin); (7) smoking; (8) drug and alcohol abuse; and (9) pregnancy or lactation [9,26].

### 2.5. Randomization and Allocation Concealment

Randomization was conducted by a statistician not involved in the study by means of a computer-generated randomization list. The allocation concealment was sealed in opaque envelopes. The envelope was opened just before the surgery and the treatment modality was revealed to the operator. The patient was blinded to the treatment allocation.

### 2.6. Surgical Intervention

All the procedures were performed by one surgeon (BG). Both sides were treated during the same appointment. After local anesthesia, the surgical area was prepared according to the modified coronally advanced tunnel technique [27]. Briefly, up to the MGJ, a full-thickness flap was prepared with a small elevator, and above the MGJ, a split-thickness flap was dissected. The papillae were detached in their buccal aspects with the periosteum. Gentle root planing was performed with a Gracey curette. In the next step, a free gingival graft was harvested from the palate and deepithelialized outside the mouth to an SCTG [28]. The length of the SCTG depended on the prepared tunnel; a width of 4 mm and a thickness of less than 1 mm was aimed for. The donor site was covered with a hemostatic collagen sponge secured with mattress sutures (Seralon 4/0 18 mm 3/8, Serag-Wiessner GmbH & Co. KG, Naila, Germany). For the test side, cross-linked hyaluronic acid (HA, hyaDENT BG, Bioscience, Germany) was applied under the flap onto the root surface according to the manufacturer’s recommendations [29]. The SCTG was inserted into the tunnel and positioned at the CEJ level with resorbable sling sutures (PGA Resorba 6/0 11 mm 3/8, RESORBA Medical GmBH, Nürnberg, Germany), and HA was applied to the entire surface of the SCTG [29]. The SCTG was fully covered with the coronally advanced flap stabilized with non-resorbable monofilament sling sutures (Seralon 6/0 12 mm 3/8, Serag-Wiessner GmbH & Co. KG, Naila, Germany). The control side was treated in the same manner, but without the HA application.

**Figure 2:**
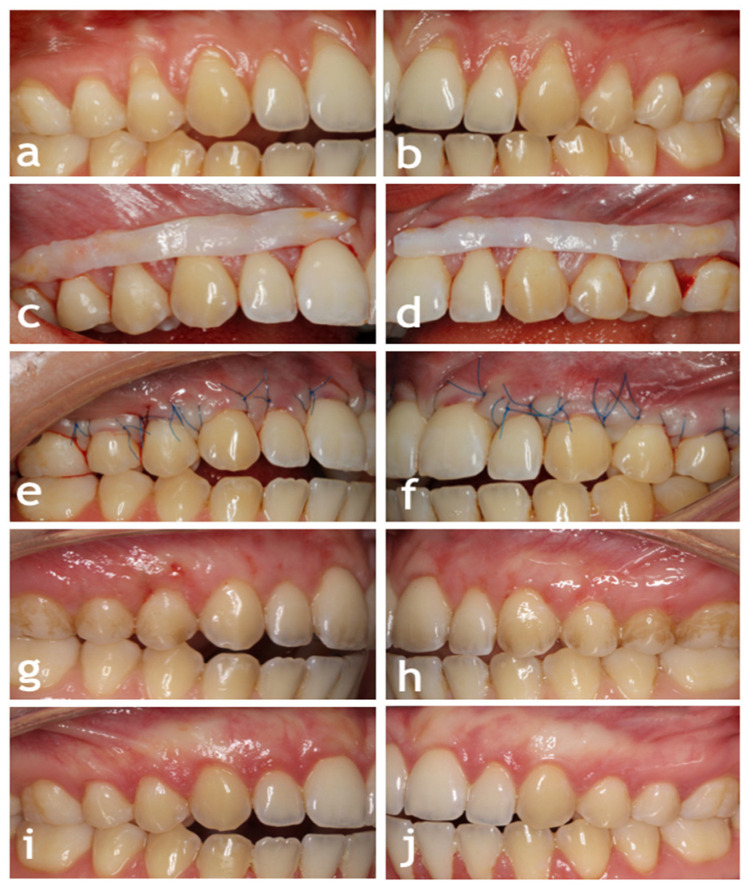
(**a**) The initial condition of gingival recessions located at teeth 16–11 on the control side. (**b**) The initial condition of gingival recessions located at teeth 21–26 on the test side. (**c**) Subepithelial connective tissue graft on the control side. (**d**) Subepithelial connective tissue graft on the test side. (**e**) Immediate postoperative view on the control side. (**f**) Immediate postoperative view on the test side. (**g**) Two-week postoperative view on the control side. (**h**) Two-week postoperative view on the test side. (**i**) Twelve-month postoperative view on the control side. (**j**) Twelve-month postoperative view on the test side.

After surgery, the patients were given 400 mg of ibuprofen and were instructed to take the second dose 8 h later [30]. They were asked to rinse twice daily with a 0.12% chlorhexidine digluconate solution for the first 3 weeks and to avoid toothbrushing and flossing. A total of 14 days after the surgery, the sutures were removed. Oral and written instructions were given to the patients. The patients were scheduled for recall appointments at 1, 3, 6, and 12 months. The clinical outcomes in one patient are shown in Figure 2.

### 2.7. Clinical Outcomes

The clinical parameters were assessed by one calibrated and blinded examiner (IS) at baseline and 12 months after the surgery. The examiner recorded the gingival recession height (GRH), recession width (RW), probing pocket depth (PPD), CAL, KTW, and GT in eight patients with at least four contralateral GRs who did not participate in the present study [9]. Two sets of measurements were performed 24 h apart. The intra-examiner intraclass correlation coefficient was 0.921 (95% CI: 0.894–0.939) for the GRH, 0.889 (0.830–0.914) for the RW, 0.902 (0.863–0.928) for the PPD, 0.893 (0.845–0.922) for the CAL, and 0.898 (0.850–0.927) for the KTW.

The following clinical measurements were recorded: (1) the gingival recession height (GRH)—the distance between the CEJ and the GM on the mid-buccal side [9]; (2) the recession width (RW)—the horizontal distance between the mesial and distal margins of the recession at the CEJ [9]; (3) the probing pocket depth (PPD)—the distance between the GM and the bottom of the pocket [9]; (4) the clinical attachment level (CAL)—the distance between the CEJ and the bottom of the pocket [9]; (5) the keratinized tissue width (KTW)—the distance between the GM and the MGJ [9]; and (6) the gingival thickness (GT), measured at the mid-buccal point of the tooth 3 mm apically from the GM [9].

A periodontal probe (PCP UNC 15; Hu-Friedy, Chicago, Illinois, USA) was used to measure the GR, RW, PPD, CAL, and KTW. The measurements were rounded down to the nearest half millimeter. The GT was evaluated using an endodontic file, 25 ISO (Poldent, Warsaw, Poland), with a stopper positioned perpendicularly to the gingiva until the surface of alveolar bone was reached. The distance between the tip of the file and the stopper was recorded using an electronic caliper with an accuracy of 0.01 (YATO YT-7201; Toya, Wrocław, Poland). The clinical parameters were registered at baseline and 12 months post-operatively.

The esthetic outcomes were assessed 12 months after the surgery by a single-blinded investigator (MS) in accordance with the RES [7,31]. The five constituents of the RES were evaluated: (1) the gingival margin (GM): 0 points—root coverage failure, 3 points—partial root coverage, 6 points—complete root coverage; (2) the marginal tissue contour (MTC): 0 points—irregular gingival contour, not following the CEJ and 1 point—scalloped gingival contour, following the CEJ; (3) the soft tissue texture (STT): 0 points—the presence of scars and 1 point—the absence of scars; (4) the mucogingival junction (MGJ): 0 points—the MGJ not aligned with the MGJ of adjacent teeth and 1 point—the MGJ aligned with the MGJ of adjacent teeth; and (5) the gingival color (GC): 0 points—the color mismatches adjacent teeth and 1 point—the color matches adjacent teeth. The ideal maximum esthetic score was 10.

### 2.8. Statistical Analysis

The results were presented using mean values, standard deviations (SD), percentages, and frequencies. The Shapiro–Wilk test confirmed that the data set was normally distributed. Consequently, Student’s *t*-test was used for comparing the means, while Pearson’s chi-square test was used to compare fractions of the test and control groups. The following parameters were calculated: (1) the mean root coverage (MRC) = GR0 – GR12/GR0 × 100%; (2) the GR reduction = GR0 – GR12; (3) the CAL gain = CAL0 – CAL 12; (4) the KTW gain = KTW12 – KTW0; and (5) the GT gain = GT12 – GT0.

To determine the predictive values of the preoperative factors for the 12-month outcomes, logistic regression models were created as follows: (1) MRC: ≤85%—low group and >85%—high group; (2) complete root coverage (CRC): binary variable; (3) RES: ≤9—low group and 10—high group; (4) KTW gain: ≤3 mm—low group and >3 mm—high group; (5) GT gain: ≤2 mm—low group and >2 mm—high group [32]. As independent variables, the HA application, tooth type (incisor/canine/premolar/molar), tooth position (maxilla/mandible), PPD, CAL, GRH, RW, KTW, GT, and recession type pre-operatively were used. All of the abovementioned parameters were analyzed with univariate logistic regression models. Next, multivariate logistic regression models were constructed using the stepwise forward technique, and V-fold cross-validation was performed, reporting the parameters of the training and testing curves. The goodness of fit was assessed by the Hosmer–Lemeshow test (where *p* > 0.05 means good fitness). Also, a multidimensional correspondence analysis was performed to confirm the relationship between the qualitative variables. The statistical significance was set at α = 0.05 and all the analyses were carried out with the Statistica Software, version 13.3 (Statsoft, Cracow, Poland).

## 3. Results

Twenty-four patients (19 females and 5 males aged between 19 and 50, with a mean age of 32.54 ± 6.67 years) with multiple symmetrical recession defects were enrolled in the study, all of whom were evaluated after 1 year. A total of 266 GRs (210 recessions in the maxilla and 56 in the mandible) were treated. Healing was uneventful and none of the patients developed any significant complications or allergic reactions. The initial characteristics of the treated sites are presented in Table 1, showing a well-balanced distribution between the treatment groups.

The mean difference for the primary endpoint was not statistically significant. After 12 months, the MRC was 84.32% for the HA + SCTG group and 85.71% for the SCTG group (*p* = 0.9910). Both groups demonstrated significant improvements in all the evaluated secondary endpoints, namely the GR reduction, CAL gain, GT gain, and KTW gain endpoints. None of the differences between the groups were statistically significant (Table 2).

Both groups achieved favorable esthetics. The overall RES values did not show statistically significant differences between the treatment arms (Table 3). The application of HA resulted in the achievement of a significantly higher value for the soft tissue texture (STT) (0.96 ± 0.20 for MCAT + HA + SCTG vs. 0.73 ± 0.22 for MCAT + SCTG, respectively).

For univariate logistic regression, the significant predictors are shown in Table 4. For both the MRC and CRC, identical results were obtained. The 12-month clinical effects of the procedure differed significantly between the types of teeth; the best results were achieved for the incisors (reference group) and the weakest were achieved for the molars (OR = 0.031, *p* = 0.001). As for the evaluated periodontal parameters, all showed significant predictive effects. The higher the values of the baseline PPD, CAL, GRH, or RW, the worse the clinical outcomes. A similar relationship was found for the GT (OR = 0.550, *p* = 0.048) and the opposite for the KTW (OR = 1.644, *p* = 0.028). For the KTW gain and GT gain, only one significantly negative prognostic factor was determined, the RW (OR = 0.477, *p* = 0.028) and the GT (OR = 0.072, *p* < 0.001) at baseline, respectively. In the context of the RES, it was found that the HA application significantly improved the 12-month clinical outcomes by nearly 2.5 times (OR = 2.477, *p* = 0.001). In this respect, both canines and molars demonstrated significantly worse effects compared to incisors. Again, a higher RW suggested significantly worse outcomes (OR = 0.813, *p* = 0.021), while a higher KTW was associated with better outcomes (OR = 1.353, pe = 0.008).

In multivariate logistic regression modelling, stepwise forward models for four parameters (MRC, GT gain, RES, and CRC) with a very good quality of fit were obtained (Table 5). Again, the results for the MRC and CRC turned out to be identical. The prognosis in terms of these parameters allowed for the best assessment of a set of periodontal variables, including the CAL, KTW, and tooth type. In the case of the GT gain, in addition to the baseline GT, the PPD was included in the model. With respect to the RES, the positive 12-month clinical outcomes were influenced by both the type of tooth (incisor or premolar) and the application of HA and a higher baseline KTW.

**Figure 3:**
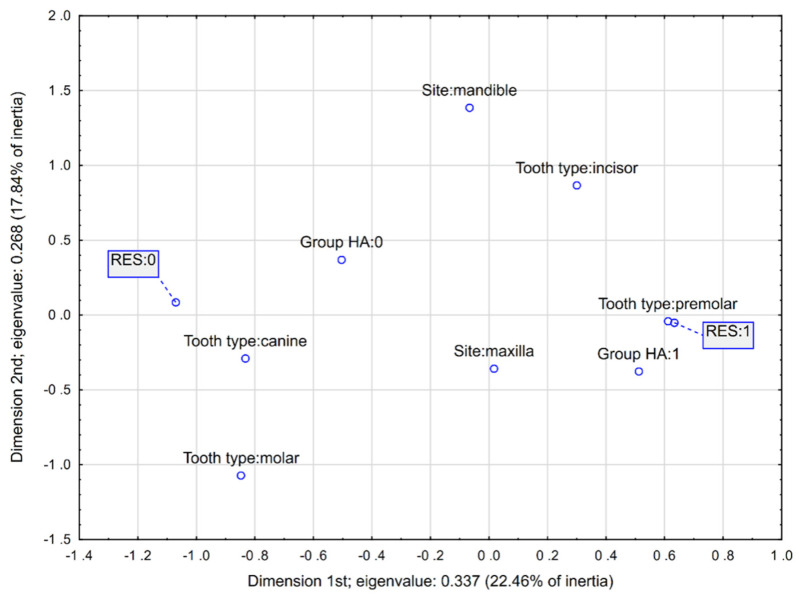
Multidimensional correspondence analysis for 12-month RES and potential qualitative predictors (Burt’s table, 10 × 10).

Additionally, a multidimensional correspondence analysis for the RES was carried out, as this was the only parameter for which the HA application was a significant predictor. Decisions on the number of MCA dimensions were made based on the scree plot. This analysis confirmed that an HA application was associated with a better 12-month RES. Also, the maxillary premolars seemed to be characterized by improved clinical outcomes (Figure 3). Detailed point parameters are reported in Table 6.

## 4. Discussion

The present RCT compared the 12-month clinical outcomes after the treatment of multiple RT1 and RT2 recessions using an MCAT with cross-linked HA (tests) or without (controls). Both treatments generated comparable clinical and esthetic results. The only statistical difference between the groups was found in the soft tissue surface characteristics, which was in favor of the test group. Customized regression models indicated that an HA application, the tooth type, the baseline CAL, and the baseline KTW were the strongest predictors for obtaining an MRC > 85%, achieving CRC, and gaining a perfect RES. To the best of our knowledge, this is the first RCT to report feasible preoperative predictors of the clinical outcomes after a surgical treatment of multiple GRs with an MCAT and HA.

The predictive impact of baseline site-related anatomical factors on the outcomes of root coverage surgery have been investigated in numerous studies. It is well documented that the larger the baseline GR height and width, the less likely CRC will be achieved [31,32,33,34]. CRC was more often observed at sites with a baseline recession depth of ≤ 2.5 mm [6]. In another study, the baseline mid-buccal CAL was significantly associated with the 12-month MRC, CRC, and RES [32]. With each 1 mm increase in the baseline CAL, the chances of positive outcomes decreased 0.32-fold in terms of the MRC, 1.29-fold for gaining CRC, and 3.42-fold for achieving a perfect RES. Our results are compatible with these findings, as the baseline CAL values were a significant negative prognostic factor for the value of the MRC.

It has previously been reported that tooth position influences the MRC and CRC [30]. Canines and incisors have been reported to achieve better outcomes compared to premolars and molars [22,35]. Moreover, maxillary teeth achieved a higher root coverage than mandibular teeth [6,36,37]. Differences in anatomical characteristics, including bigger papillae in the upper arch, the presence of lip muscles, and a minor vestibular depth in the lower arch were used to explain the abovementioned observations. The present multilevel model analysis demonstrated that tooth type might be a significant prognostic factor for the MRC, CRC, and the RES. The highest MRC and CRC were achieved for the incisors, whereas the premolars were associated with optimal RES values. The molars had the highest odds of 12-month failure. On the other hand, the present study revealed no significant effect of tooth position (upper versus lower arch) on the treatment outcomes. Our outcomes are consistent with the findings of a very recent meta-analysis in which the overall MRC of the tunnel technique for multiple maxillary GR defects was 88.63 ± 7.08%, while the ARC for multiple mandibular GR defects was 85.88 ± 27.77% [6]. This observation may be explained, at least in part, by the characteristics of the SCTG that was used. The extraoral de-epithelialization of free gingival grafts allows connective tissue to be harvested directly from under the epithelium, which is stable and of optimal biological potential [28].

It goes without saying that tooth location should always be evaluated together with site-specific parameters, such as the KTW and GT. The mean KTW and GT values at baseline were previously correlated with statistically significant positive effects on the MRC (*p* = 0.004 and *p* = 0.013, respectively) after a multiple GR treatment with the MCAT [34]. In other studies, the KTW was pinpointed as the main factor in obtaining root coverage with an MCAT, and the accepted consensus is that a KTW of about 2 mm is desirable [38,39,40]. The baseline GT value was a significantly positive prognostic factor for the MRC after an MCAT + SCTG [34,41]. The required minimum mean soft tissue thickness to achieve CRC increased from 1.44 mm (12 months) to 1.61 mm (24 months) [41]. With each 1 mm increase in the baseline GT, the probability of achieving CRC increased tenfold [32]. Consequently, by integrating the KTW and the gingival thickness, clinical guidance was enhanced [27,39]. In the present study, the baseline KTW, but not the GT, independently predicted the 12-month MRC and CRC. Our results support the statement that graft material might play a crucial role in terms of the clinical outcomes, as an SCTG was placed in all the treated recessions independently of the KTW or GT values. It was suggested by Cairo [42] that a graft behaves as a biological filler under the surgical flap, hence decreasing postoperative shrinkage and improving the recession treatment outcomes.

The final soft tissue quality coupled with the esthetic results may be considered the most important treatment goal of root coverage procedures. Apart from CRC, other factors related to natural soft tissue appearance play a crucial role in the final esthetics. While 60% of the RES value is due to CRC, the remaining 40% is determined by the marginal soft tissue contour, the soft tissue texture, the presence of keloids, the gingival color, and the muco-gingival junction alignment. In the present study, both groups achieved favorable esthetic outcomes, which is in line with data from previously published studies and the conclusions of recent systematic reviews and meta-analyses [6,7]. It is also noteworthy that the test sides were found to achieve a better soft tissue texture. Furthermore, the multilevel regression and multidimensional correspondence analyses revealed that the additional application of HA increased the likelihood of a perfect RES by more than twofold. The variance in this parameter could be related to the regenerative properties of HA on cells involved in soft tissue wound healing. On the histological level, HA promoted a moderate increase in collagen fiber density and a significant improvement in the elastic fiber network in biopsy samples taken from the sides treated with an SCTG + HA when compared to the sides treated only with an SCTG [23]. It was also reported that the use of HA might reduce soft tissue scarring. Asparuhova et al. [43] demonstrated that HA enhanced the expression of genes encoding type III collagen and transforming growth factor-β3, characteristic of scarless wound healing. At the final phase of the healing process, HA interacts with CD44 and a receptor for HA-mediated motility (RAHMM) to promote the maturation of collagens (type I) for extracellular matrix remodeling [44]. However, the reasons for this finding are open to speculation. According to some recent studies, the site-specific application of an SCTG was suggested to further enhance the esthetic outcome. Even though SCTG procedures showed the highest overall esthetic performance for root coverage, the graft integration may impede the natural soft tissue color and appearance [7]. By the same token, an SCTG should be selectively positioned under the surgical flap in sites presenting with a thin phenotype or a reduced band of KTW < 2 mm [39,40]. It has been speculated that the addition of a graft in the presence of a thick gingiva may result in a bulky appearance of the soft tissues.

Even though this RCT evaluated only local risk predictors, systemic and environmental factors such as age, gender, and tobacco consumption have been previously associated with the extent of gingival recessions [45]. GRs occur in populations with both high and low standards of oral hygiene [46]. Males used to have more GRs than females, but gender was not significant risk indicator in all the evaluated populations [45,46,47,48]. These discrepancies may be related to methodological differences between studies, as different variables were included in the multivariate analysis. All in all, men had worse oral hygiene habits, and were more likely to ignore their oral health and engage in preventive dental care less often than women [49]. Women, on the other hand, exhibited a higher oral health literacy, better oral health behaviors, more positive attitudes, and a greater adherence to dental treatment [50]. Consequently, gender-related factors might influence treatment planning and patient management in clinical practice.

Within the limitations of the present investigation, it should be mentioned that only a single formulation of HA was used, thus creating methodological bias. Furthermore, the recruitment of patients with multiple and symmetrical GRs may have led to selection bias. RT3 recessions were not included in this study, as these defects do not show predictable root coverage [3]. Third, as all the surgeries were performed by a single, trained clinician, external validity was probably limited. Last, but not least, the sample size was based on the only available clinical trial at the time, but with a different surgical modality [24]. Despite the limitations cited above, this is the first RCT to assess the surgical treatment of multiple GRs using an MCAT in combination with HA and an SCTG; thus, no direct comparisons with other studies were feasible. The strengths of this study include the approaches used to maintain scientific rigor by following the CONSORT guidelines. The data were collected by calibrated and blinded examiners, and all the surgeries were performed by one experienced surgeon. The final sample size amounted to 24 patients and 266 GRs. Taking into account the statistically significant effect size, the findings of this RCT can be considered solid. In addition to the main purpose of this study, it would be interesting to follow these patients and evaluate the long-term outcomes. Nevertheless, to draw more definitive conclusions and extend the findings of the present RCT, further research projects and evidence are required. Future research should focus on optimizing the doses and delivery methods to optimize the therapeutic potential of HA. Given the technical challenges inherent to the procedure, the results need to be confirmed on a wider basis.

## 5. Conclusions

Within its limitations, this study demonstrated that the use of HA in addition to an MCAT + SCTG for the soft tissue augmentation of multiple RT1 and RT2 recessions improved the 12-month esthetic outcomes. The baseline CAL, the KTW, and the tooth type predicted the 12-month MRC, CRC, and RES. An assessment of preoperative factors might help clinicians in the decision-making process for multiple GR treatments.

## Figures and Tables

**Table 1:** Initial characteristics of the test and control groups.

Variables	Test Group (SCTG + HA) (N = 24, n = 133)	Control Group (SCTG) (N = 24, n = 133)
Gender (n) Women Men	19 5	19 5
Tooth type (n) Incisors Canines Premolars Molars	42 23 45 23	42 23 45 23
Tooth position (n) Maxillary teeth Mandibular teeth	108 25	105 28
Type of GR (n,%) RT1 RT2	59 (44%) 74 (56%)	54 (41%) 79 (59%)

GR—gingival recession; HA—hyaluronic acid; N—number of patients; n—number of recessions; RT—recession type; SCTG—subepithelial connective tissue graft.

**Table 2:** Clinical parameters (mean + SD) at baseline and 12 months post-operative.

	Baseline	12 Months Post-Operative	*p* (Baseline–1 Year)
MRC for SCTG + HA MRC for SCTG *p* (test vs. control)		84.32 ± 34.46 85.71 ± 36.43 0.9910	
CRC for SCTG + HA CRC for SCTG *p* (test vs. control)		92.12 ± 28.14 94.61 ± 24.71 0.8994	
GRH for SCTG + HA GRH for SCTG *p* (test vs. control)	1.77 ± 1.13 1.67 ± 1.12 0.8121	0.12 ± 0.48 0.08 ± 0.39 0.9983	<0.0001 <0.0001
GR red for SCTG+HA GR red for SCTG *p* (test vs. control)		1.65 ± 1.09 1.59 ± 1.14 0.9205	<0.0001 <0.0001
RW for SCTG + HA RW for SCTG *p* (test vs. control)	3.24 ± 1.8 3.32 ± 1.82 0.9881	0.35 ± 1.29 0.25 ± 1.09 0.2332	<0.0001 <0.0001
PPD for SCTG + HA PPD for SCTG *p* (test vs. control)	1.42 ± 0.54 1.49 ± 0.57 0.3112	1.42 ± 0.53 1.49 ± 0.54 0.7811	0.9982 0.4837
CAL for SCTG + HA CAL for SCTG *p* (test vs. control)	3.08 ± 1.28 3.08 ± 1.22 0.9801	0.50 ± 0.85 0.57 ± 0.80 0.8993	<0.0001 <0.0001
KTW for SCTG + HA KTW for SCTG *p* (test vs. control)	2.80 ± 1.38 2.69 ± 1.28 0.8911	3.57 ± 1.49 3.57 ± 1.26 0.8938	0.2092 0.2100
KTW gain for SCTG + HA KTW gain for SCTG *p* (test vs. control)		0.68 ± 1.40 0.76 ± 1.36 0.5882	
GT for SCTG + HA GT for SCTG *p* (test vs. control)	1.68 ± 0.72 1.70 ± 0.75 0.9278	2.54 ± 0.74 2.54 ± 0.67 0.9992	0.0351 0.0388
GT gain for SCTG + HA GT gain for SCTG *p* (test vs. control)		0.81 ± 0.79 0.77 ± 0.74 0.7862	

CAL—clinical attachment level; CRC—complete root coverage; GRH—gingival recession height; GR red - gingival recession reduction; GT—gingival thickness; HA—hyaluronic acid; KTW—keratinized tissue width; MRC—mean root coverage; PPD—probing pocket depth; RW—recession width; SCTG—subepithelial connective tissue graft.

**Table 3:** Esthetic outcomes (mean + SD) 12 months after surgery.

	GM	MTC	STT	MGJ	GC	RES
SCTG + HA SCTG *p* (test vs. control)	5.75 ± 0.83 5.78 ± 0.87 0.9491	0.90 ± 0.30 0.87 ± 0.34 0.9271	0.96 ± 0.20 0.73 ± 0.22 0.0091	0.92 ± 0.28 0.89 ± 0.31 0.7703	0.98 ± 0.14 0.98 ± 0.13 0.9981	9.51 ± 1.01 9.26 ± 1.10 0.7292

GC—gingival color; GM—gingival margin; HA—hyaluronic acid; MGJ—mucogingival junction alignment; MTC—marginal tissue contour; RES—root coverage esthetic score; SCTG—subepithelial connective tissue graft; STT—soft tissue texture.

**Table 4:** Significant predictors in univariate logistic regression modelling.

Treatment Outcome	Predictor	Category or Unit	OR [95% CI]	*p*
MRC 85%	Tooth type	incisor canine premolar molar	reference 0.313 [0.028–3.565] 0.597 [0.053–6.730] 0.031 [0.004–0.249]	0.350 0.676 0.001
	PPD	1 mm	0.378 [0.160–0.893]	0.027
	CAL	1 mm	0.348 [0.199–0.609]	<0.0001
	GRH	1 mm	0.569 [0.380–0.852]	0.006
	RW	1 mm	0.458 [0.335–0.626]	<0.0001
	KTW	1 mm	1.644 [1.054–2.565]	0.028
	GT	1 mm	0.550 [0.304–0.995]	0.048
CRC	Tooth type	incisor canine premolar molar	reference 0.313 [0.028–3.565] 0.597 [0.053–6.730] 0.031 [0.004–0.249]	0.350 0.676 0.001
	PPD	1 mm	0.378 [0.160–0.893]	0.027
	CAL	1 mm	0.348 [0.199–0.609]	<0.001
	GRH	1 mm	0.569 [0.380–0.852]	0.006
	RW	1 mm	0.458 [0.335–0.626]	<0.001
	KTW	1 mm	1.644 [1.054–2.565]	0.028
	GT	1 mm	0.550 [0.304–0.995]	0.048
RES	HA	no yes	reference 2.477 [1.433–4.282]	0.001
	Tooth type	incisor canine premolar molar	reference 0.347 [0.158–0.765] 1.210 [0.589–2.486] 0.341 [0.151–0.768]	0.009 0.603 0.009
	RW	1 mm	0.813 [0.682–0.969]	0.021
	KTW	1 mm	1.353 [1.081–1.693]	0.008
KTW gain	RW	1 mm	0.447 [0.201]	0.048
GT gain	GT	1 mm	0.072 [0.019–0.275]	<0.0001

CAL—clinical attachment level; CI—confidence interval; CRC—complete root coverage; GRH—gingival recession height; GT—gingival thickness; HA—hyaluronic acid; KTW—keratinized tissue width; MRC—mean root coverage; OR—odds ratio; PPD—probing pocket depth; RES—root coverage esthetic score; RW—recession width; SCTG—subepithelial connective tissue graft.

**Table 5:** Multivariate models based on stepwise forward logistic regression.

Model	Treatment Outcome	Goodness of Fit (*p*-Value for Hosmer–Lemeshow)	Predictor	Category or Unit	OR [95% CI]	*p*
Model I	MRC 85%	0.618	Tooth type	incisor canine premolar molar	reference 0.560 [0.045–6.968] 0.964 [0.080–11.568] 0.046 [0.005–0.389]	0.652 0.977 0.005
			CAL	1 mm	0.396 [0.202–0.776]	0.007
			KTW	1 mm	1.698 [1.004–2.872]	0.048
Model II	CRC	0.618	Tooth type	incisor canine premolar molar	reference 0.560 [0.045–6.968] 0.964 [0.080–11.568] 0.046 [0.005–0.389]	0.652 0.977 0.005
			CAL	1 mm	0.396 [0.202–0.776]	0.007
			KTW	1 mm	1.698 [1.004–2.872]	0.048
Model III	RES	0.743	HA	no yes	reference 2.683 [1.495–4.814]	0.001
			Tooth type	incisor canine premolar molar	reference 0.402 [0.173–0.936] 1.570 [0.720–3.425] 0.374 [0.158–0.885]	0.035 0.257 0.025
			KTW	1 mm	1.378 [1.070–1.776]	0.013
Model IV	GT gain	0.239	GT	1 mm	0.055 [0.013–0.231]	<0.0001
			PPD	1 mm	3.271 [1.318–8.119]	0.011

CAL—clinical attachment level; CI—confidence interval; CRC—complete root coverage; GRH—gingival recession height; GT—gingival thickness; HA—hyaluronic acid; KTW—keratinized tissue width; MRC—mean root coverage; OR—odds ratio; PPD—probing pocket depth; RES—root coverage esthetic score; RW—recession width; SCTG—subepithelial connective tissue graft.

**Table 6:** Detailed parameters of determined points in multidimensional correspondence analysis for 12-month RES and potential qualitative predictors.

	x	y	Quality	Relative Inertia	x Inertia	x cos^2^	y Inertia	y cos^2^
Group: SCTG + HA	−0.503	0.370	0.397	0.083	0.095	0.258	0.065	0.139
Group: SCTG	0.512	−0.377	0.397	0.084	0.097	0.258	0.066	0.139
Tooth type: incisor	0.300	0.867	0.345	0.118	0.019	0.037	0.204	0.308
Tooth type: canine	−0.833	−0.289	0.180	0.135	0.097	0.161	0.015	0.019
Tooth type: premolar	0.612	−0.042	0.203	0.108	0.097	0.202	0.001	0.001
Tooth type: molar	−0.848	−1.071	0.385	0.138	0.091	0.148	0.183	0.237
Site: maxilla	0.017	−0.358	0.496	0.034	0.0001	0.001	0.095	0.495
Site: mandible	−0.066	1.385	0.496	0.132	0.001	0.001	0.368	0.495
RES: 0	−1.070	0.085	0.682	0.105	0.316	0.678	0.003	0.004
RES: 1	0.633	−0.050	0.682	0.062	0.187	0.678	0.001	0.004

HA—hyaluronic acid; RES—root coverage esthetic score; SCTG—subepithelial connective tissue graft.

## Data Availability

The data presented in this study are available from the corresponding author upon request.

## References

[1] Wennström J.L. (1996). Mucogingival therapy. Ann. Periodontol..

[2] Albandar J.M., Kingman A. (1999). Gingival recession, gingival bleeding, and dental calculus in adults 30 years of age and older in the United States, 1988–1994. J. Periodontol..

[3] Cairo F., Nieri M., Cincinelli S., Mervelt J., Pagliaro U. (2011). The inter-proximal clinical attachment level to classify gingival recessions and predict root coverage outcomes: An explorative and reliability study. J. Clin. Periodontol..

[4] Zucchelli G., Mounssif I. (2015). Periodontal plastic surgery. Periodontol. 2000.

[5] Chambrone L., Botelho J., Machado V., Mascarenhas P., Mendes J.J., Avila-Ortiz G. (2022). Does the subepithelial connective tissue graft in conjunction with a coronally advanced flap remain as the gold standard therapy for the treatment of single gingival recession defects? A systematic review and network meta-analysis. J. Periodontol..

[6] Tavelli L., Barootchi S., Nguyen T.V.N., Tattan M., Ravidà A., Wang H.L. (2018). Efficacy of tunnel technique in the treatment of local- ized and multiple gingival recessions: A systematic review and meta-analysis. J. Periodontol..

[7] Cairo F., Barootchi S., Tavelli L., Barbato L., Wang H.L., Rasperini C., Graziani F., Tonetti M. (2020). Aesthetic- and patient-related outcomes following root coverage procedures: A systematic review and network meta-analysis. J. Clin. Periodontol..

[8] Gobbato L., Nart J., Bressan E., Mazzocco F., Paniz G., Lops D. (2016). Patient morbidity and root coverage outcomes after the application of a subepithelial connective tissue graft in combination with a coronally advanced flap or via a tunneling technique: A randomized controlled clinical trial. Clin. Oral Investig..

[9] Aroca S., Molnár B., Windisch P., Gera I., Salvi G.E., Nikolidakis D., Sculean A. (2013). Treatment of multiple adjacent Miller class I and II gingival recessions with a modified coronally advanced tunnel (MCAT) technique and a collagen matrix or palatal connective tissue graft: A randomized, controlled clinical trial. J. Clin. Periodontol..

[10] Azaripour A., Kissinger M., Farina V.S., Van Noorden C.J., Gerhold-Ay A., Willershausen B., Cortellini P. (2016). Root coverage with connective tissue graft associated with coronally advanced flap or tunnel technique: A randomized, double-blind, mono-centre clinical trial. J. Clin. Periodontol..

[11] Mayta-Tovalino F., Barboza J.J., Pasupuleti V., Hernandez A.V. (2023). Efficacy of Tunnel Technique (TUN) versus Coronally Advanced Flap (CAF) in the Management of Multiple Gingival Recession Defects: A Meta-Analysis. Int. J. Dent..

[12] Ozenci I., Ipci S.D., Cakar G., Yilmaz S. (2015). Tunnel technique versus coronally advanced flap with acellular dermal matrix graft in the treatment of multiple gingival recessions. J. Clin. Periodontol..

[13] Cieślik-Wegemund M., Wierucka-Młynarczyk B., Tanasiewicz M., Gilowski Ł. (2016). Tunnel technique with collagen matrix compared with connective tissue graft for treatment of periodontal recession: A randomized clinical trial. J. Periodontol..

[14] Górski B., Górska R., Wysokińska-Miszczuk J., Kaczyński T. (2020). Tunnel technique with enamel matrix derivative in addition to subepithelial connective tissue graft compared with connective tissue graft alone for the treatment of multiple gingival recessions: A randomized clinical trial. Clin. Oral Investig..

[15] Korkmaz B., Balli U. (2021). Clinical evaluation of the treatment of multiple gingival recessions with connective tissue graft or concentrated growth factor using tunnel technique: A randomized controlled clinical trial. Clin. Oral Investig..

[16] Ferguson E.L., Roberts J.L., Moseley R., Griffiths P.C., Thomas D.W. (2011). Evaluation of the physical and biological properties of hyaluronan and hyaluronan fragments. Int. J. Pharm..

[17] Tavianatou A.G., Caon I., Franchi M., Piperigkoum Z., Galesso D., Karamanos N.K. (2023). Hyaluronian: Molecular size-dependent signaling and biological functions in inflammation and cancer. FEBS J..

[18] Iaconisi G.N., Lunetti P., Gallo N., Cappello A.R., Fiermonte G., Dolce V., Capobianco L. (2023). Hyaluronic Acid: A powerful biomolecule with wide-ranging applications—A comprehensive review. Int. Mol. J. Sci..

[19] Manfredini M., Beretta M., Maiorana C., Tandurella M., Federica E.S., Poli P.P. (2023). Effectiveness of adjunctive hyaluronic acid application in surgical treatment of gingival recession sites. Prosthesis.

[20] Oates T.W., Robinson M., Gunsolley J.C. (2003). Surgical therapies for treatment of gingival recession. A systematic review. Ann. Periodontol..

[21] De Sanctis M., Clementini M. (2014). Flap approaches in plastic periodontal and implant surgery: Critical elements in design and execution. J. Clin. Periodontol..

[22] Tonetti M.S., Jepsen S. (2014). Clinical efficacy of periodontal plastic surgery procedures: Consensus Report of Group 2 of the 10th European Workshop on Periodontology. J. Clin. Periodontol..

[23] Skierska I., Górski B., Fus Ł. (2024). Tunnel technique and subepithelial connective tissue graft, with or without cross-linked hyaluronic acid, in the treatment of multiple gingival recessions: 12-month outcomes of a randomized clinical trial. J. Periodontol..

[24] Nandanwar J., Bhongade M.L., Puri S., Dhadse P., Datir M., Kasatwar A. (2018). Comparison of effectiveness of hyaluronic acid in combination with polylactic acid/polyglycolic acid membrane and subepi-thelial connective tissue graft for the treatment of multiple gingival recession defects in human: A clinical study. J. Datta Meghe Inst. Med. Sci. Univ..

[25] Chambrone L., Tatakis D.N. (2015). Periodontal soft tissue root coverage procedures: A systematic review from the AAP Regeneration Workshop. J. Periodontol..

[26] Pilloni A., Schmidlin P.R., Sahrmann P., Sculean A., Rojas M.A. (2019). Effectiveness of adjunctive hyaluronic acid application in coronally advanced flap in Miller class I single gingival recession sites: A randomized controlled clinical trial. Clin. Oral Investig..

[27] Sculean A., Cosgarea R., Stähli A., Katsaros C., Arweiler N.B., Miron R.J., Deppe H. (2016). Treatment of multiple adjacent maxillary Miller Class I, II, and III gingival recessions with the modified coronally advanced tunnel, enamel matrix derivative, and subepithelial connective tissue graft: A report of 12 cases. Quintessence Int..

[28] Zucchelli G., Mele M., Stefanini M., Mazzotti C., Marzadori M., Montebugnoli L., de Sanctis M. (2010). Patient morbidity and root coverage outcome after subepithelial connective tissue and de-epithelialized grafts: A comparative randomized-controlled clinical trial. J. Clin. Periodontol..

[29] Lanzrein C., Guldener K., Imber J.C., Katsaros C., Stähli A., Sculean A. (2020). Treatment of multiple adjacent recessions with the modified coronally advanced tunnel or laterally closed tunnel in conjunction with cross-linked hyaluronic acid and subepithelial connective tissue graft: A report of 15 cases. Quintessence Int..

[30] Giorgetti A.P.O., Matos R., Casarin R.C.V., Pimentel S.P., Cirano F.R., Ribeiro F.V. (2018). Preemptive and Postoperative Medication Protocols for Root Coverage Combined with Connective Tissue Graft. Braz. Dent. J..

[31] Cairo F., Rotundo R., Miller P.D., Pini Prato G.P. (2009). Root coverage esthetic score: A system to evaluate the esthetic outcome of the treatment of gingival recession through evaluation of clinical cases. J. Periodontol..

[32] Górski B., Górska R., Szerszeń M., Kaczyński T. (2022). Modified coronally advanced tunnel technique with enamel matrix derivative in addition to subepithelial connective tissue graft compared with connective tissue graft alone for the treatment of multiple gingival recessions: Prognostic parameters for clinical treatment outcomes. Clin. Oral Investig..

[33] Xue F., Zhang R., Liu J., Duan J., Zhang Y., Cai Y. (2023). Digitally measured exposed root surface area for predicting the effectiveness of modified coronally advanced tunnel combined de-epithelialized gingival grafting in the treatment of multiple adjacent gingival recessions. Clin. Oral Investig..

[34] Bakhishov H., Isler S.C., Bozyel B., Yıldırım B., Tekindal M.A., Ozdemir B. (2021). De-epithelialized gingival graft versus subepithelial connective tissue graft in the treatment of multiple adjacent gingival recessions using the tunnel technique: 1-year results of a randomized clinical trial. J. Clin. Periodontol..

[35] Zucchelli G., Tavelli L., Ravidà A., Stefanini M., Suárez-López Del Amo F., Wang H.L. (2018). Influence of tooth location on coronally advanced flap procedures for root coverage. J. Periodontol..

[36] Aroca S., Barbier A., Clementini M., Renouard F., de Sanctis M. (2018). Treatment of class III multiple gingival recessions: Prognostic factors for achieving a complete root coverage. J. Clin. Periodontol..

[37] Pietruska M., Skurska A., Podlewski Ł., Milewski R., Pietruski J. (2018). Clinical evaluation of Miller class I and II recessions treatment with the use of modified coronally advanced tunnel technique with either collagen matrix or subepithelial connective tissue graft: A randomized clinical study. J. Clin. Periodontol..

[38] Kim D.M., Neiva R. (2015). Periodontal soft tissue non-root coverage procedures: A systematic review from the AAP Regeneration Workshop. J. Periodontol..

[39] Rasperini G., Codari M., Limiroli E., Acunzo R., Tavelli L., Levickiene A.Z. (2019). Graftless Tunnel Technique for the Treatment of Multiple Gingival Recessions in Sites with Thick or Very Thick Biotype: A Prospective Case Series. Int. J. Periodontics Restor. Dent..

[40] Aroca S., Di Domenico G.L., Darnaud C., de Sanctis M. (2021). Modified Coronally Advanced Tunnel Technique with Site-Specific Application of Connective Tissue Graft for the Treatment of Multiple Adjacent Maxillary Gingival Recessions: A Case Series. Int. J. Periodontics Restor. Dent.

[41] Zuhr O., Rebele S.F., Vach K., Petsos H., Hürzeler M.B., Research Group for Oral Soft Tissue Biology & Wound Healing (2020). Tunnel technique with connective tissue graft versus coronally advanced flap with enamel matrix derivative for root coverage: 2-year results of an RCT using 3D digital measuring for volumetric comparison of gingival dimensions. J. Clin. Periodontol..

[42] Cairo F. (2017). Periodontal plastic surgery of gingival recessions at single and multiple teeth. Periodontol..

[43] Asparuhova M.B., Kiryak D., Eliezer M., Mihov D., Sculean A. (2019). Activity of two hyaluronan preparations on primary human oral fibroblasts. J. Periodontal. Res..

[44] Yang H., Song L., Zou Y., Sun D., Wang L., Yu Z., Guo J. (2021). Role of Hyaluronic Acids and Potential as Regenerative Biomaterials in Wound Healing. ACS Appl. Bio Mater..

[45] Sarfati A., Bourgeois D., Katsahian S., Mora F., Bouchard P. (2010). Risk assessment for buccal gingival recession defects in an adult population. J. Periodontol..

[46] Cortellini P., Bissada N.F. (2018). Mucogingival conditions in the natural dentition: Narrative review, case definitions, and diagnostic considerations. J. Periodontol..

[47] Kassab M.M., Cohen R.E. (2003). The etiology and prevalence of gingival recession. J. Am. Dent. Assoc..

[48] Susin C., Haas A.N., Oppermann R.V., Haugejorden O., Albandar J.M. (2004). Gingival recession: Epidemiology and risk indicators in a representative urban Brazilian population. J. Periodontol..

[49] Lipsky M.S., Su S., Crespo C.J., Hung M. (2021). Men and Oral Health: A Review of Sex and Gender Differences. Am. J. Men’s Health.

[50] Furuta M., Ekuni D., Irie K., Azuma T., Tomofuji T., Ogura T., Morita M. (2011). Sex differences in gingivitis relate to interaction of oral health behaviors in young people. J. Periodontol..

